# Pandemic influenza preparedness and health systems challenges in Asia: results from rapid analyses in 6 Asian countries

**DOI:** 10.1186/1471-2458-10-322

**Published:** 2010-06-08

**Authors:** Piya Hanvoravongchai, Wiku Adisasmito, Pham Ngoc Chau, Alexandra Conseil, Joia de Sa, Ralf Krumkamp, Sandra Mounier-Jack, Bounlay Phommasack, Weerasak Putthasri, Chin-Shui Shih, Sok Touch, Richard Coker

**Affiliations:** 1 Cambodia Ministry of Health, Department of Communicable Disease Control, 151-153 Kampuchea Ground Avenue, Phnom Penh, Cambodia; 2 Department of Health Policy & Administration, Faculty of Public Health, University of Indonesia, Depok 16424, Indonesia; 3 National Emerging Infectious Diseases Coordination Office, Fa Ngoum Road, Vientiane, Lao PDR; 4 Taiwan Centers for Disease Control, No.6, Linsen S. Rd., Jhongjheng District, Taipei City 10050, Taiwan; 5 International Health Policy Programme-Thailand, Ministry of Public Health, Nonthaburi 11000, Thailand; 6 Vietnam Military Medical University, Hanoi, Vietnam; 7 Department of Health Sciences, Faculty of Life Sciences, Hamburg University of Applied Sciences, Lohbrügger Kirchstr. 65, 21033 Hamburg, Germany; 8 Communicable Diseases Policy Research Group, Department of Public Health and Policy, London School of Hygiene & Tropical Medicine, Keppel Street, London WC1E 7HT, UK

## Abstract

**Background:**

Since 2003, Asia-Pacific, particularly Southeast Asia, has received substantial attention because of the anticipation that it could be the epicentre of the next pandemic. There has been active investment but earlier review of pandemic preparedness plans in the region reveals that the translation of these strategic plans into operational plans is still lacking in some countries particularly those with low resources. The objective of this study is to understand the pandemic preparedness programmes, the health systems context, and challenges and constraints specific to the six Asian countries namely Cambodia, Indonesia, Lao PDR, Taiwan, Thailand, and Viet Nam in the prepandemic phase before the start of H1N1/2009.

**Methods:**

The study relied on the Systemic Rapid Assessment (SYSRA) toolkit, which evaluates priority disease programmes by taking into account the programmes, the general health system, and the wider socio-cultural and political context. The components under review were: external context; stewardship and organisational arrangements; financing, resource generation and allocation; healthcare provision; and information systems. Qualitative and quantitative data were collected in the second half of 2008 based on a review of published data and interviews with key informants, exploring past and current patterns of health programme and pandemic response.

**Results:**

The study shows that health systems in the six countries varied in regard to the epidemiological context, health care financing, and health service provision patterns. For pandemic preparation, all six countries have developed national governance on pandemic preparedness as well as national pandemic influenza preparedness plans and Avian and Human Influenza (AHI) response plans. However, the governance arrangements and the nature of the plans differed. In the five developing countries, the focus was on surveillance and rapid containment of poultry related transmission while preparation for later pandemic stages was limited. The interfaces and linkages between health system contexts and pandemic preparedness programmes in these countries were explored.

**Conclusion:**

Health system context influences how the six countries have been preparing themselves for a pandemic. At the same time, investment in pandemic preparation in the six Asian countries has contributed to improvement in health system surveillance, laboratory capacity, monitoring and evaluation and public communications. A number of suggestions for improvement were presented to strengthen the pandemic preparation and mitigation as well as to overcome some of the underlying health system constraints.

## Background

"World 'well prepared' for virus" is the title of a news article from the BBC on 27 April 2009, a day the World Health Organization (WHO) raised the level of influenza pandemic alert from Phase 3 to Phase 4 [1]. The article cited a high-level WHO officer who commented that "the international community is better prepared than ever" to handle the potential influenza pandemic, because several years of preparation for avian flu had helped countries build up stockpiles of antiviral drugs globally. On the same day, a spokesman for the WHO Regional Office for the Western Pacific declared that "Asia is better prepared and in a better position than others" citing experience in management of and response to the Severe Acute Respiratory Syndrome (SARS) outbreak which affected the Region in 2003 [2].

Having established a large antiviral stockpile and/or having experience with SARS does not necessarily mean that a country is well equipped to face an influenza pandemic. Preparedness is a complex phenomenon which involves many aspects, including disease surveillance, case management, command and control, and community containment [3]. Earlier studies on the completeness of national pandemic influenza preparedness plans in several regions reveal that many challenges and important gaps in preparedness remain [4-9]. Besides, these studies show that the level of preparedness varies hugely across and within regions. The situation in developing countries is the most worrisome as their public health infrastructure is often weak with severe shortage in financial, human, and technical resources [7,10-12].

Since 2003, Asia-Pacific, particularly Southeast Asia, has received substantial attention because of the anticipation that it could be the epicentre of the next pandemic. There has been active investment in preparedness strategy and planning in many countries by both domestic and international players. Despite such strong interest and investment, a review of strategic pandemic preparedness plans in Asia in 2006 and a report on regional preparedness published by the United Nations System Influenza Coordinator (UNSIC) in 2007 reveals that the translation of these strategic plans into operational plans is still lacking in many countries in the region [4,13].

This paper presents the results from a rapid situation analysis (RSA) of health system and pandemic preparedness in six countries of the Asia-Pacific region prior to the H1N1/2009 epidemic. Taiwan had extensive experience with the SARS outbreak, with over 300 confirmed cases. Viet Nam, Thailand, and Indonesia also had SARS cases (albeit fewer than Taiwan) and, together with Lao PDR and Cambodia, have had human Avian Influenza cases. Besides, endemicity of the influenza subtype H5N1 is found in poultry in these five countries.

The objectives of this rapid situation analysis are to describe the pandemic preparedness programmes and the health systems context in which these programmes have been established, and to identify challenges and constraints specific to the six countries. It is a part of a bigger project, the Asia*FluCap *project, which aims to evaluate health system capacity in these countries in response to different phases of influenza pandemic. The study was conducted in the second half of 2008 with funding support from the European Union and the Rockefeller Foundation.

## Methods

**Figure 1:**
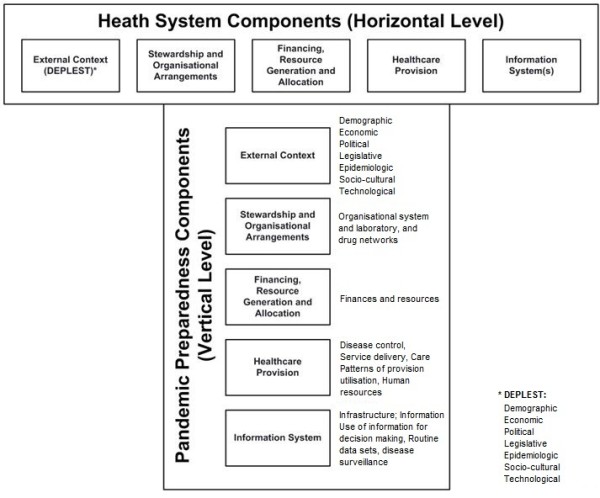
**Components of the Systemic Rapid Assessment Toolkit (SYSRA)**.

This study relies on the Systemic Rapid Assessment (SYSRA) Toolkit which is a systematic approach for gathering information about structures and modes of operation from complex health systems [14]. It builds on the SYSRA Framework, a conceptual and analytical framework initially developed by Atun et al. to evaluate health systems and communicable disease control programmes [15,16]. The SYSRA analytical framework provides a conceptual, analytical framework and tool to evaluate health interventions that takes into account disease programmes, the general health system, and the wider socio-cultural and political context. For the purpose of this study, this framework was adapted to pandemic influenza. Our SYSRA toolkit comprises of two core elements: (i) the 'health systems element' and (ii) the 'pandemic preparedness programme element' (Figure 1). The health systems element focuses on structures and functionality of an overall health system (horizontal level). The 'pandemic preparedness programme element' assesses the specific pandemic influenza programme components embedded within a health system (vertical level). For each element, the components under review are: external context; stewardship and organisational arrangements; financing, resource generation and allocation; healthcare provision; and information systems.

The study was conducted in the second half of 2008. For each of the RSA modules qualitative and quantitative data were collected based on a review of published data, documentation and interviews with key informants in each country. As a first step, secondary data and documentation was reviewed and summarised for each country in order to determine what information was available and what data was lacking. Afterwards, an interview team consisting of members (public health specialists) familiar with the health system and pandemic management programme in the country conducted interviews with key informants using a predefined semi-structured questionnaire, focusing especially on gaps identified in the initial literature review. The questions explored past and current patterns of health programme response, changes in pandemic response and other historical information about outbreak management. While conducting the interviews, additional qualitative and quantitative information were collected through an iterative process of information gathering.

Key informants were selected on the basis of their expertise in a broad range of health system and pandemic programme components. They were chosen from all administrative levels and from different institutions to provide a triangulated view of the health system and of the pandemic management programme. Field visits occurred between October to December 2008 with up to 21 key informants being interviewed in each country. No remuneration was provided to the informants. The lists of field researchers and the affiliations and roles of the key informants from each country are provided in the Country Case Study Reports available publicly accessible on the website: http://www.cdprg.org/publications. Ethical approval for this project was obtained from all participating countries.

The scope of this study is limited to health system and health service response and preparation for pandemic influenza. Non-health sector preparation and mitigation efforts are beyond the scope of this analysis. The choice of the six countries is based on an historical assessment that they would likely be at the epicentre of an influenza pandemic, the incidence of H5N1 in poultry, and their experience with SARS.

## Results

### Context and health system characteristics

The country contexts in the six study countries vary considerably. The political systems include republic (Taiwan and Indonesia), constitutional monarchy (Thailand and Cambodia), and socialist republic (Vietnam and Lao PDR). The level of economic development based on World Bank's classification ranges from low income with high agricultural share (Lao PDR, Cambodia, and Viet Nam), middle income (Thailand and Indonesia) to industrialized and high income (Taiwan). There is, however, similarity in that all countries enjoyed relative political stability (except recently in Thailand) and continuous economic growth over the past decade preceding the current global economic crisis.

Health systems in the six countries vary in regard to the current health status and epidemiological profile, the level of health care resource, health financing mechanisms and health service provision patterns (Table 1).

**Table 1:** Selected indicators of health system contexts in 6 study countries.

	Cambodia		Indonesia		Lao PDR		Taiwan		Thailand		Viet Nam	
Population (in thousands)	14,197		228,864		5,759		22,880		63,444		86,206	

Human Development Index*	0.575		0.726		0.608		0.93	^2005^	0.786		0.718	

GDP per capita (current US$)	512	^#^	1,592	^#^	591	^#^	16,740	^&^	3,258	^#^	708	^#^

Adult literacy rate (%)	73.6	^2004^	90.4	^2004^	68.7	^2001^	96.1	^2003&^	92.6	^2000^	90.3	^1999^

Life expectancy at birth (years) both sexes	62		68		60		77.89		72		72	

Hospital beds (per 10,000 population)	1	^2004^	5.2	^2005^^	12	^2005^	57.3	^^^	21.4	^2005^^	26.6	

Physicians density (per 10,000)	2	^2000^	1	^2003^	4	^2004^	16	^2007^	4	^2000^	6	^2002^

Nursing and midwifery personnel density (per 10,000)	9	^2000^	8	^2003^	10	^2004^	43.1	^2007^	28	^2000^	8	^2002^

Total expenditure on health as percentage of GDP	6		2.2		3.6		6.1		3.5		6.6	

Per capita total expenditure on health (US$)	30		34		22		970	^2005^	113		46	

Skilled birth attendance (%)	44		66		19		N/A		97		88	

Note: Data are from WHO http://www.who.int/whosis/ for the year 2006 unless specify otherwise;

*HDI from UNDP Human Development Report; #GDP per Capita from World Bank's World Development Indicators;

^^ ^Hospital bed density for Taiwan from Department of Health, for Indonesia from Public Health Expenditure Review 2008 by the World Bank, and for Thailand from Thailand Health Profile 2005-7;

^&^GDP per capita and Adult literacy rate for Taiwan are from CIA World Fact Book.

• *Current health status and epidemiological profile*

Taiwan shows a pattern of industrialized economies post epidemiological transition with low mortality, high life expectancy, and high disease burden from chronic diseases. In contrast to Taiwan, Lao PDR and Cambodia have lower life expectancy with high morbidity and mortality from communicable diseases.

• *Level of healthcare resources*

The level of health system resources reflects the level of economic development. Taiwan has a high level of health spending and high density of hospital beds and health workforce per capita. On the other end of the spectrum, Cambodia and Lao PDR have low health spending and very low health facility and health workforce density. External resources are a significant source of health financing in Cambodia and Lao PDR.

• *Health financing mechanisms*

Only Taiwan and Thailand have universal coverage of health insurance. Indonesia and Viet Nam have a number of health insurance schemes such as social security scheme and government employee health insurance for different sectors of the population. Lao PDR and Cambodia relied mainly on out-of-pocket payments with recent development of community financing. Lao PDR is developing social security insurance.

• *Health service provision*

Health service provision patterns in the six countries are mixed. The private sector plays a major role in Taiwan. In both Thailand and Viet Nam, the public sector has an extensive network of public health facilities. However, a significant proportion of population is increasingly using private sector health care providers such as drug stores and private clinics as their first source of health care. In Indonesia, Lao PDR, and Cambodia, the availability of health facilities is quite limited as seen in the density of hospital beds which is at 1 per 1,000 or less. One indicator of health service access is the proportion of skilled birth attendance. The statistics in 2006 shows that the proportion was over 80% in Thailand and Viet Nam, around two-third in Indonesia, nearly 50% in Cambodia and less than 20% in Lao PDR in 2006.

### Pandemic preparedness programme

All countries in this study have experienced an outbreak of SARS or avian influenza in humans (Table 2). During the SARS outbreak, Taiwan was severely affected with 346 casualties. There were 63, nine, and two confirmed cases in Vietnam, Thailand and Indonesia respectively [17]. For Avian and Human Influenza (AHI), more than 100 human cases have been reported each in Viet Nam and Indonesia, 25 in Thailand, eight in Cambodia, and two in Lao PDR. There were no AHI cases in Taiwan.

**Table 2:** SARS and AHI Cases.

	Cambodia	Indonesia	Lao PDR	Taiwan	Thailand	Viet Nam
Total SARS Cases (reported probable cases)*	0	2	0	665	9	63

Total Human AHI Cases^#^	8	141	2	0	25	110

Total Human Deaths from AHI^#^	7	115	2	0	17	55

Case Fatality Ratio (Deaths/All Cases) ^#^	88%	82%	100%	-	68%	50%

Provinces with Human AHI Cases/Total Provinces	5 of 24	13 of 33	1 of 17	0 of 25	18 of 76	32 of 64

Note: *from WHO: Summary table of SARS cases by country, 1 November 2002 - 7 August 2003 http://www.who.int/csr/sars/country/2003_08_15/en/index.html;

^#^Cumulative Number of Confirmed Human Cases of Avian Influenza A/(H5N1) Reported to WHO as of 23 April 2009 http://www.who.int/csr/disease/avian_influenza/country/en/index.html

All six countries have developed national governance on AHI and pandemic preparedness. They all have national pandemic preparedness plans and AHI response plans. However, the governance arrangements and the nature of the plans differ across the countries. Moreover, the operational procedures as well as strategic directions vary. This section presents the preparedness arrangements in regard to governance and stewardship, financial resources, other resources, and health service provision in the study period.

• *Governance & Stewardship*

In all countries, the pandemic preparedness committees were headed by the president or prime minister or his/her representative. In Indonesia, Lao PDR, and Thailand the national pandemic preparedness plans and the AHI response plans were integrated together with AHI response plan as a part of pandemic preparedness plan. The other countries had separated plans for pandemic preparedness from the AHI response plans.

At the central/national level, there were three main patterns of pandemic preparedness governance. First, as in Lao PDR and Indonesia, a special coordination unit (National AHI Coordination Office (NAHICO) which recently changed its name to National Emerging Infectious Diseases Coordination Office (NEIDCO) in Lao PDR, and National Committee for AI Control and Pandemic Preparedness (KOMNAS) for Indonesia) was established specifically to coordinate AHI and pandemic related activities as a priority programme (vertical policy approach). Second, in Vietnam, the governance relies on existing governance structure e.g. responsible agencies only. Third, in Cambodia, Taiwan and Thailand, pandemic preparedness is situated as part of programmes on disaster preparedness and mitigation so the preparation for pandemic is framed within the national disaster response.

There was also a difference in the governance in regard to the level of responsibility. This reflects the existing governance structure and the nature of devolution of governing power in the country. In Cambodia, Lao PDR, Taiwan, Thailand, where resource allocation decisions are centralized, the budget allocation towards AHI and pandemic preparedness programmes was also decided mostly at central level. In Indonesia and Viet Nam, central authority was important but local authorities also played crucial roles in the decision and priority setting of the level of pandemic preparedness investment in their regions. Nevertheless, in all countries the operational activities of pandemic preparation at the local level were allocated to/integrated within the network of existing government bodies.

• *Financial resource*

Data on government and external spending for AHI and preparedness are not readily available and our best estimate shows that most countries spent around 1 USD per capita per year or less on these activities (Table 3). Whereas the level of to the disease was highest in 2006 and 2007, it declined in 2008. Funding solely or mostly originated from central budget, except in Indonesia and Viet Nam where the local source of funding was also important, and to a lesser extent in Thailand. All countries but Cambodia, had discretionary budget for local level administration to use on AHI and pandemic preparation.

**Table 3:** Estimated AHI and Pandemic preparedness Budget in Million USD (USD per capita in parenthesis).

	2004	2005	2006	2007	2008
Cambodia*			0.034 (0.002)	1.48 (0.1)	3.02 (0.2)

Lao PDR			11.44 (2.0)	2.1 (0.4)	6.42 (1.1)

Indonesia			55.0 (0.2)	93.8 (0.4)	6.1 (0.0)

Taiwan	14.5 (0.6)	24.5 (1.1)	53.0 (2.3)	57.0 (2.5)	22.5 (1.0)

Thailand^#^		35 (0.6)	58 (0.9)	44 (0.7)	

Viet Nam^#^		82.2 (1.0)			

Note: * only budget for ADB CDC Regional Project; ^# ^Government budget only

External resources have been substantial for low income countries, particularly Lao PDR and Cambodia. Almost the entire budget for AHI and pandemic preparedness activities in Lao PDR and Cambodia was provided by external donors and international organizations. Indonesia also drew in a significant amount of external funding for AHI and pandemic preparedness, accounting for almost one-fourth of total budget. There was no external financial support for Taiwan and less than 3 percent in Thailand. No data was available for Vietnam.

• *Other resources: human resource, vaccine, drugs, technology*

In all six countries, pandemic preparedness activities at the operational level relied on existing healthcare workforce in the public sector. Hence human resources available for AHI are reflective of the health workforce situation in public health system. Shortage of highly skilled workers was a major problem in all developing countries, especially in relation to physicians and nurses. In regard to specific knowledge and skills for pandemic influenza, additional trainings were provided to specific sections of the workforce in all countries, particularly to those working in surveillance, case detection, and infection control. Most, except Taiwan and Viet Nam, did not have a plan for surge capacity of health care workers during pandemic time. Moreover, there is a question over potential absenteeism among existing workforce at the time of pandemic.

All countries have strengthened their laboratory investigation capacity to prepare for the potential pandemic. All, except Lao PDR, had Biosafety Level 3 (BSL 3) laboratory capacity and can conduct virus sequencing. These five countries were also capable of immuno-fluorescence assay (IFA) and reverse transcription- polymerase chain reaction (RT-PCR). Only Taiwan had the capacity to produce pre-pandemic vaccine and has a plan to increase its capacity towards pandemic vaccine production by 2010. Indonesia, Thailand, Viet Nam had plans, or were in the process of conducting research, towards developing their pandemic vaccine production capacity. Taiwan, Thailand, and Indonesia had local capacity to produce antiviral drugs from chemical entities.

All countries had stockpiles of antivirals and personal protective equipment (PPE) but the size of the stockpiles varied across countries. In Taiwan, the national stockpile was enough to treat 10% of population and there is a plan to increase this stockpile if necessary. The national stockpiles of Thailand and Indonesia covered approximately 1% of their population while in Cambodia the national stock in Phnom Penh was enough for 0.1% of the population (15,780 doses). We were unable to estimate the size of the stockpiles in Lao PDR and Viet Nam from key informants or reviewed documents. In most countries, the antiviral stockpiles were located at both central level and at hospital and local health authorities. In addition to national stockpiles, there was an ASEAN regional stockpile in Singapore.

• *Health service*

Health service preparedness for pandemic influenza highly concentrated on surveillance and rapid containment activities in all countries but Taiwan. The surveillance systems were mainly facility and community based surveillance systems where suspected cases are reported to the central level authority for further investigation and containment. Several channels for case reporting have been set up including telephone hotline, SMS, email and websites. All countries except Lao PDR also conducted laboratory surveillance of samples from influenza-like-illness cases.

The surveillance system for pandemic influenza in the five countries with history of AHI focused on poultry related cases. When there were animal cases of avian influenza in the neighbourhood, patients with influenza-like illness with history of poultry contacts would be specially monitored. In these countries active collaboration between human and animal health sectors to conduct joint surveillance was reported. Also, Surveillance Rapid Response Teams (SRRTs) have been set up at both central level and local level based on existing capacity, to be readily available for field investigation when there is a suspected case. In countries with shortage of qualified human resources, the surveillance and response capacity at local level remains a major challenge. Only Taiwan and Viet Nam had explicit plans for surveillance and response in time of pandemic.

All countries have assigned referral hospitals to take care of AHI cases in the pre-pandemic phases. A model hospital preparedness plan has been developed in most countries to be used by their health facilities in time of pandemic. Hospital surge capacity (extra beds) has been planned in all countries but Lao PDR and Cambodia. Similar to surveillance and response, only Taiwan and Viet Nam had an explicit staff surge capacity plan. Lao PDR and Taiwan had additional plans to use volunteer in time of pandemic.

In regard to case management, the focus was mainly on AHI cases. Clinical treatment guidelines for AHI infection have been developed in all countries. Training on clinical management of AHI cases has been conducted with patient isolation and antiviral treatment as the main instruments. In all countries there was a policy to provide antiviral prophylaxis to AHI contacts. However, there was no clear rationing policy on antiviral distribution in case of pandemic. All countries (except Taiwan which has not reported any case) have provided free care to all AHI patients thus far.

In the five countries where human cases have been reported, most infected patients arrived at hospital after their symptoms had developed for several days. In these countries, a patient generally seeks self medication or informal/private primary-care providers as his/her first contact point and only visit public health facilities when the symptoms are severe. This is compounded by the relative lack of health care facilities in lower resource countries like Cambodia and Lao and the high use of private care facilities in Cambodia.

There were active public health education efforts in all countries. In the countries with AHI cases, most of the messages and materials were related to the handling of livestock and basic health hygiene such as hand washing, protection when sneezing/coughing. The main strategy of public health education was to focus on the prevention of Avian Influenza transmission (e.g. use chicken as a mascot, etc). Very few messages were on pandemic influenza.

A number of simulation exercises have been conducted in all six countries. Most of the exercises were table-top style where relevant officers discuss and manage a hypothetical pandemic situation in a round-table manner. For example, Thailand had at least one table-top exercise at the central level and in each province. Viet Nam has conducted many simulations for AHI preparedness at national, provincial and district level as well as at airport and borders. There were also a few regional (multi-country) table-top exercises coordinated by the World Health Organization and one table-top exercise by the Mekong Basin Disease Surveillance Network (MBDS). Only Indonesia and Taiwan had full-scale exercises involving real field activities. Indonesia's full-scale exercise in Bali in April 2008 was the first of its kind in the world. Taiwan's full-scale exercise at its national airport focused on its response to the arrival by plane of a suspected H5N1 case. Most exercises reveal that management and coordination between various players, including non-health sector players, constitutes a major weakness in preparedness. A criticism common to all six countries is that most simulations exercises have focused on early containment but not on pandemic preparedness in later phases.

The preparation for mitigation efforts at more advanced stages of a pandemic was quite limited in most countries. They have identified various channels for risk communication to the public. However, only Taiwan had clear operation procedures to sustain service provision and resource mobilization when widespread pandemic occurs. The researchers also found that knowledge/skills for pandemic preparation at local level were more limited than central level staff.

## Discussion

The rapid analyses in six Asian countries show a strong link between the health system functions and pandemic preparation. In all countries, the health system context shapes how pandemic preparedness in the country is carried out. From the RSA we found that the interfaces/linkages between health system contexts and pandemic preparedness programmes are particularly strong in three areas: governance and stewardship, resources, and service provision.

The arrangements and strength of **governance and stewardship **of pandemic preparedness programme follow those of the general health system. In well-established health systems, pandemic preparedness is integrated within existing mechanisms, notably within the national disaster preparedness framework. In countries with a weak healthcare system, new vertical programme had to be established to manage and coordinate pandemic preparedness and response.

The nature of pandemic governance also depends on the existing political context. Decentralized countries have greater challenges to deal with during both outbreaks and pandemics. In a decentralized system like in Indonesia, the level of political commitment could affect the level of investment in pandemic preparedness in that region/area as seen in the contrasting difference between Bali and Jakarta. In Jakarta, where political interest on pandemic is low, the planned table-top simulation exercise was postponed because of the lack of budget while in Bali, a full-scale exercise was carried out with strong support from all sectors.

The political and historical context also shapes the pandemic preparedness process. For example, the political crisis in Thailand in 2008 resulted in frequent changes of Minister of Public Health and several postponements of national pandemic preparedness committee meetings. In Taiwan, pandemic preparation is high on national political agenda because of its previous history of SARS outbreak and casualties as well as a perceived threat of bioterrorism.

The level of **resource **available for pandemic preparedness depends on the level of economic and health system development of the country. The amount of financial investment in preparedness activities and stockpiling of drugs and equipments is dependent on the level of budget availability. Countries with low financial resource need to rely on external funding for their pandemic preparedness activities. The series of H5N1 outbreaks which have occurred in the region since 2003 combined to the heightened global interests in averting a pandemic have allowed many low resource countries to draw in financial resources to support their preparation especially for surveillance and early detection. However, there are questions about the sustainability of these external resources given the current global economic recession and other public health priorities in donor countries themselves. Such resources might also be much more difficult to mobilize during pandemic time. Similarly, the shortage of highly skilled workers in the general health system has been raised as a major limitation of the preparedness planning and response in many of these countries. This situation could be even more serious in pandemic time when a number of staff may become ill with the disease and some of them may be absent due to the fear of infection.

**Health service provision **for AHI control relies primarily on the existing provider system. The main strategy used in all countries but Taiwan is to focus on early detection and containment. Investment was made into rapid response team and surveillance mechanisms with attention to the linkages between poultry infection and human cases. This strategy may be driven by several factors. The emergence of human cases of H5N1 may have led each of the five countries to strongly assume that outbreaks of human-to-human transmission could start within their own country. Moreover, the potential threat of the H5N1 pandemic also drew external funding whose main interest may have been to rapidly contain avian influenza outbreaks within the region, hence investment in surveillance and case detection. Besides, the lack of internal resources may have yield to limited investment in pharmaceutical interventions such as antiviral and vaccine stockpiling. The WHO Pandemic classification system into various phases could have also influenced countries into investing first in preparedness for the earlier phases and to delay preparedness for the later phases, although phases will remain fluid during a pandemic as the H1N1/2009 has demonstrated.

Investment in pandemic preparedness activities has contributed to the strengthening of health system functions in many countries specifically in regards to surveillance, laboratory capacity, monitoring and evaluation, and public communication. Regionally, there has been active cooperation through the surveillance network in the Mekong basin through the Mekong Basin Disease Surveillance network (MBDS). These health system functions could be useful for other diseases beyond pandemic response. However, the low investment in clinical care in relation to other health services may be a big challenge for these countries, especially if a pandemic is to expand beyond the early containment phase.

### Improving pandemic preparedness and health system strengthening

The outbreak of influenza H1N1/2009 and its spread globally also raises many important questions on how prepared these Asian countries are for global pandemic influenza. The underlying assumption that the pandemic would start from avian influenza virus mutation within the country led to heavy investment on surveillance and case detection mechanisms in the five developing countries. These mechanisms were designed primarily for AHI with reliance on poultry contact history in the surveillance and case detection operational guidelines and unlikely to be effective for early-detection and containment of pandemic influenza now that human-to-human transmission has been observed without an animal tracer. The pandemic response strategy and the surveillance and case detection protocols in these countries need to be transformed to accommodate this changing circumstance. It is also important to translate existing pandemic response and mitigation plans into operations particularly at the subnational level as local administration and communities need to be active and ready for these plans to be effective.

Limited stockpiles of the antivirals, covering 1% or less of the population in all countries other than Taiwan, raise the issue of drug allocation when a large-scale high-impact pandemic occurs. The World Health Organization recommended countries to stockpile antivirals for 20% of their population but this is obviously not feasible financially for many developing countries [10,12]. Similarly, it is already clear with the H1N1/2009 outbreak that when the pandemic vaccine is developed its availability will be limited [18]. Explicit rationing or prioritization policy for the medicines and vaccines is necessary and should be developed to avoid ethical and political conflicts that may arise [19-21].

The ongoing threat of pandemic influenza with human-to-human transmission also calls for a revision/reposition of public education campaigns that were shown to be focusing on animal to human transmission in many Southeast Asian countries. The message requires adjustment from current emphasis on animal handling hygiene to respiratory health hygiene and when to seek medical care. The current treatment strategy to rely on a referral hospital system may also need to be adjusted towards community level surge capacity and the use of volunteers to support the system in time of pandemic. Simulation exercises with phase 6 hypothetical scenarios could be useful as a test of the level of preparedness especially with actors from non-health sector.

For the preparation to be effective and sustainable, the interventions need not only focus on the influenza related activities. Our study shows that health systems provide important context towards the success of the responses. The effort to strengthen pandemic preparedness should also be done in such a way that also strengthens health systems. Three areas of improvement based on our findings of strong linkages between pandemic preparation and health systems in governance and stewardship, health system resource, and service provision are highlighted here.

Firstly, the governance and stewardship of AHI and pandemic preparedness should be integrated into the broader disaster preparedness system. Taiwan benefited from more resources from higher level of economic development but comprehensive and multisectoral responses with commitment from all levels also resulted from high political interest and a systematic approach to preparedness using disaster and bioterrorism response system. National ownership of the preparedness activities is particularly important especially in low resource countries where external funding is prominent. The allocation decision of pandemic related investment should be harmonised and aligned with national systems and priorities.

Secondly, the scarcity of health care resources particularly in rural areas was shown to hamper the preparation for the pandemic as well as the responses to other diseases. Scaling up health system capacity such as health workforce and health care infrastructure is necessary and should be decided based on evidence together with effective planning. For example, the countries can benefit from the Asia*FluCap *project's ongoing analysis of health system resource gaps to effectively respond to pandemic. Nevertheless, investment in health workforce and health care infrastructure should avoid disease-specific focus and contribute to overall system strengthening [22]. A number of tools and proposed actions for scaling up disease specific capacity that also promote health system strengthening are increasingly available [22-24].

Lastly, in service provision the preparedness strategy also needs to address the prominent role of the private sector. Private providers are the first contact point for health care in many countries. In many countries where the linkage of information system between public and private sector does not exist, the surveillance system may not be able to detect the cases early enough before it has already spread. Treatment success could also be lower and the fatality rate could be higher if the patients present themselves late to public health care system where antiviral medicines are prescribed. The pandemic and disaster responses could also tap into the capacity of private non-profit network and volunteers to support the scaling up of necessary responses. Better planning and coordination between public and private sector health providers and is necessary and should be strengthened.

### Study limitations

This study contains a number of limitations. First, the rapid nature of the analysis was useful for simplicity, speed, and limited cost but it also limits the extent and the depth of the analyses. This limitation is alleviated by the way the questionnaires and data collection procedures were designed. Published and grey literature documents were reviewed prior to and after field visits to prepare and verify the data received from the interviews.

Second, there are potential biases from key informants' selection. These were mitigated by including resource persons from different health system levels and sectors to allow for the triangulation of results from various sources. Additionally, the data collection including interviews was carried out by both external and local experts to balance the views and to provide systematic, robust, contextual understanding.

Third, the scope of the analysis is limited to pandemic influenza and the health systems. Other competing health care needs and priorities were assessed to a limited extent in the analysis of health care context. Relative importance of those needs could influence how health systems respond to pandemic influenza, which could add to the complexity of the analysis. Additionally, a pandemic could create adverse social events beyond health impacts and interrupts essential services such as food logistics or water and electricity supply systems. Our study did not explore multisectoral responses or the continuity of essential services beyond the health sector, which is important and deserves further careful evaluation.

Additional research should be conducted to shed more light into pandemic preparation in these Asian countries. A number of research activities are now going on as part of the Asia*FluCap *project. These include the analyses of health system capacity and resource distribution in the country, scenario development for identification of resource requirements at different stages of a pandemic, and stakeholder analyses to better understand the political context and relationship between actors. Future research may include the implications of pandemic preparedness on health systems e.g. financial trend, health workforce burden, the economic analyses of resource needed to fill the capacity gaps, and so on.

## Conclusion

The study in late 2008 prior to the H1N1/2009 epidemic shows that the health system context influences how the six countries have been preparing themselves for a pandemic. The level and form of pandemic preparedness depend on existing health systems arrangements particularly its governance, resource, and existing service provision patterns. The political and historical context of previous epidemics shaped the priority given to pandemic preparation in a country. Countries with limited domestic resources rely heavily on external funding for pandemic preparation activities. The fragmentation of health information and referral systems in some countries particularly in relation to linkage with private sector providers constitutes a challenge in synergistic pandemic response.

Pandemic preparation in the six Asian countries has contributed to improvement in health system surveillance, laboratory capacity, monitoring and evaluation and public communications. However, preparation for pandemic mitigation in countries with low health system resources is still rather limited. With the emergence of H1N1/2009, the previous preparation in the five developing countries based on the AHI model of poultry to human transmission became less relevant. If a pandemic is to expand beyond the early containment phase it will be a big challenge for these countries whether their health system will have enough capacity to effectively respond.

A number of suggestions for improvement were presented to strengthen the pandemic preparation and mitigation as well as to overcome three areas of the underlying health system constraints - governance and stewardships, resources, and service provision. The heightened public interest and awareness on the ongoing pandemic could be mobilized towards more investment in health systems.

## Competing interests

RC has received funding from F Hoffmann-La Roche, various governments, and the European Commission.

## Authors' contributions

Design of the study: RC, SMJ, RK. Data collection and analyses: JdS, PH, SMJ, AC, RK, RC, WA, PNC, BP, WP, CSS, ST. Manuscript writing: PH, AC, SMJ, RC. All authors contributed to the revision of the draft manuscript. All authors read and approved the final manuscript.

## Pre-publication history

The pre-publication history for this paper can be accessed here:

http://www.biomedcentral.com/1471-2458/10/322/prepub

## References

[B1] BBC News World 'well prepared' for virus 2009 http://news.bbc.co.uk/1/hi/world/americas/8019566.stm accessed on 27 April 2009

[B2] AFP: SWINE FLU Asia 'better prepared' to tackle outbreak WHO 2009 http://www.channelnewsasia.com/stories/afp_asiapacific/view/425205/1/.html accessed on 27 April 2009

[B3] World Health Organization WHO checklist for influenza pandemic preparedness planning WHO/CDS/CSR/GIP/2005.4 2005 Geneva, World Health Organization

[B4] Coker RJ Mounier-Jack S Pandemic influenza preparedness in the Asia-Pacific region Lancet 2006 368 886 9 10.1016/S0140-6736(06)69209-X 16950366 PMC7123339

[B5] Mounier-Jack S Coker RJ How prepared is Europe for pandemic influenza? Analysis of national plans Lancet 2006 367 9520 1405 1411 10.1016/S0140-6736(06)68511-5 16650650

[B6] Mounier-Jack S Coker RJ Progress and shortcomings in European national strategic plans for pandemic influenza Bulletin of the World Health Organization 2007 85 12 10.2471/BLT.06.039834 PMC2636305 18278251

[B7] Ortu G Mounier-Jack S Coker RJ Pandemic influenza preparedness in Africa is a profound challenge for an already distressed region: analysis of national preparedness plans Health Policy Plan 2008 23 3 161 169 10.1093/heapol/czn004 18381384 PMC7314001

[B8] Mensua A Mounier-Jack S Coker RJ Pandemic influenza preparedness in Latin America: analysis of national strategic plans Health Policy Plan 2009 in press 19411447 10.1093/heapol/czp019 PMC7313980

[B9] Hong Kong SARS Expert Committee Summary Report of the SARS Expert Committee 2003 http://www.sars-expertcom.gov.hk/english/reports/reports.html accessed on 31st May 2009

[B10] Oshitani H Kamigaki T Suzuki A Major issues and challenges of influenza pandemic preparedness in developing countries Emerg Infect Dis 2008 14 6 875 80 10.3201/eid1406.070839 18507896 PMC2600290

[B11] Lee K Fidler D Avian and pandemic influenza: Progress and problems with global health governance Global Public Health 2007 2 3 215 234 10.1080/17441690601136947 19283625

[B12] Fedson DS Meeting the Challenge of Influenza Pandemic Preparedness in Developing Countries Emerg Infect Dis 2009 15 3 365 371 10.3201/eid1503.080857 19239746 PMC2681116

[B13] Brad Herbert Associates Coordination of avian and human influenza activities. A report produced for the UN System Influenza Coordinator Brad Herbert Associates 2007

[B14] Mounier-Jack SM A toolkit for rapid assessment of health systems and pandemic influenza preparedness and response: Systemic Rapid Assessment Toolkit (SYSRA) London, London School of Hygiene and Tropical Medicine 2008 http://www.cdprg.org/publications.php

[B15] World Health Organization Guide to Rapid Assessment and Response (TG-RAR) Geneva, World Health Organization http://www.who.int/hiv/pub/prev_care/tgrar/en/

[B16] Atun RA Lennox-Chhugani N Drobniewski F Samyshkin YA Coker RJ A framework and toolkit for capturing the communicable disease programmes within health systems tuberculosis control as an illustrative example EJPH 2004 14 267 73 10.1093/eurpub/14.3.267 15369032

[B17] World Health Organization Summary of probable SARS cases with onset of illness from 1 November 2002 to 31 July 2003 http://www.who.int/csr/sars/country/table2004_04_21/en/accessed on 31st May 2009

[B18] UK Department of Health Agreements secured for pre-pandemic vaccine for the UK 2009 http://webarchive.nationalarchives.gov.uk/+/www.dh.gov.uk/en/Publichealth/Flu/Swineflu/DH_099248 accessed on 31st May 2009

[B19] Straetemans M Buchholz U Reiter S Haas W Krause G Prioritization strategies for pandemic influenza vaccine in 27 countries of the European Union and the Global Health Security Action Group: a review BMC Public Health 2007 7 236 10.1186/1471-2458-7-236 17825095 PMC2048949

[B20] Uscher-Pines L Omer SB Barnett DJ Burke TA Balicer RD Priority Setting for Pandemic Influenza: An Analysis of National Preparedness Plans PLoS Med 2006 3 10 e436 10.1371/journal.pmed.0030436 17048982 PMC1609123

[B21] Arino J Bowman CS Moghadas SM Antiviral resistance during pandemic influenza: implications for stockpiling and drug use BMC Infectious Diseases 2009 9 8 10.1186/1471-2334-9-8 19161634 PMC2653495

[B22] Travis P Bennett S Haines A Pang T Bhutta Z Hyder A Pielemeier N Mills A Evans T Overcoming health-systems constraints to achieve the Millennium Development Goals Lancet 2004 364 900-06 10.1016/S0140-6736(04)16987-0 15351199

[B23] Task Force on Human Resources for Health Financing What Countries Can Do Now: Twenty-Nine Actions to Scale-Up and Improve the Health Workforce 2009 World Health Organization, Geneva http://www.who.int/workforcealliance/knowledge/publications/taskforces/actionpaper.pdf

[B24] Mangham L Hanson K Scaling up in international health: what are the key issues? Health Policy & Planning 2010 25 2 10.1093/heapol/czp066 20071454

